# Management of post-cesarean pelvic abscess in the uterine scar: A case report and mini-review of the literature

**DOI:** 10.3892/mi.2026.323

**Published:** 2026-05-18

**Authors:** Efthymia Thanasa, Anna Thanasa, Emmanouil Xydias, Ioannis Paraoulakis, Evangelos Kamaretsos, Apostolos Ziogas, Ioannis Thanasas

**Affiliations:** 1 Department of Health Sciences, Medical School, Aristotle University of Thessaloniki, 54124 Thessaloniki, Greece; 2 Department of Obstetrics and Gynaecology, EmbryoClinic IVF of Thessaloniki, 55133 Thessaloniki, Greece; 3 Department of Obstetrics and Gynecology, General Hospital of Trikala, 42100 Trikala, Greece; 4 Third Department of Obstetrics and Gynecology, University General Hospital ‘Attikon’, Medical School, National and Kapodistrian University of Athens, 12462 Athens, Greece; 5 Department of Medicine, University of Thessaly, School of Health Sciences, 41334 Larissa, Greece

**Keywords:** cesarean section, uterine scar, pelvic abscess, ultrasound, computed tomography scan, treatment, laparotomy

## Abstract

Cesarean section is among the most frequently performed surgical procedures worldwide. In recent years, the increasing rate of cesarean deliveries has led to an increased incidence of post-operative complications. The present case report describes a rare case of a post-operative pelvic abscess located at the uterine scar following a cesarean section, managed successfully via laparotomy. The patient, a 17-year-old woman, was admitted 1 week after undergoing a cesarean delivery. She presented with abdominal pain, fever and chills. Blood tests revealed elevated inflammatory markers and a mild coagulation disorder, consistent with an ongoing infectious process. The results of urinalysis did not reveal any notable findings. A transvaginal ultrasound and an abdominal computed tomography scan revealed a large, mixed echogenic mass with irregular borders and the presence of intralesional air bubbles, localized at the uterine scar anterior to the bladder. Given the persistence of clinical symptoms, supportive imaging and laboratory findings, and the failure of conservative management of the post-operative intra-abdominal infection, surgical intervention was deemed necessary. A laparotomy was performed through the previous cesarean incision. Intraoperatively, an abscess was identified at the site of the uterine scar and was surgically drained. Samples were collected for aerobic and anaerobic cultures, and the cavity was irrigated thoroughly to remove purulent and hemorrhagic material. A drainage tube was placed before closure. The postoperative course of the patient was uneventful, with complete clinical resolution. Following the case presentation, a brief review of the current diagnostic and therapeutic strategies for this uncommon post-operative complication is also provided. On the whole, the present case report underscores the importance of timely surgical intervention via laparotomy when image-guided drainage or laparoscopy is not feasible.

## Introduction

Cesarean section is among the most commonly performed surgical procedures worldwide, with >25 million procedures conducted annually (1,2). The global rate of primary cesarean delivery is estimated at 21.1%, and the proportion of women with previous cesarean sections undergoing scheduled repeat procedures ranges from 80 to 90%. This ongoing rise in cesarean births has contributed to an increased incidence of post-operative complications (3). The most frequent and potentially life-threatening complication requiring surgical reintervention is primary or secondary postpartum hemorrhage. Other severe, albeit less common, complications include pelvic hematoma or abscess, puerperal sepsis, internal bleeding and uterine wound dehiscence, all of which carry a significant risk of maternal morbidity and mortality (4).

Pelvic abscess formation at the site of a uterine scar following a cesarean section is a rare, yet severe complication (5). Common anatomical locations for such abscesses include the space between the broad ligaments, the posterior cul-de-sac (Douglas pouch), and the vesicouterine space (6), as observed in the case discussed in the present report. Several risk factors have been associated with the development of post-operative pelvic abscesses, including young maternal age, low socioeconomic status, obesity, prolonged labor with cephalopelvic disproportion and multiple vaginal examinations, the premature rupture of membranes, an unscheduled cesarean delivery, and a history of prior cesareans complicated by extensive intraperitoneal adhesions (7). In the case presented herein, the cesarean section was elective and performed prior to the onset of labor. The patient was not obese [body mass index (BMI), 26.7] and did not belong to a low socioeconomic group. The only notable risk factor identified was her young maternal age (17 years).

The present case report describes a case of pelvic abscess located at the uterine scar following cesarean section, successfully managed through laparotomy. A brief discussion is provided on modern diagnostic and therapeutic approach to this rare post-operative complication, emphasizing the need for timely reoperation when other approaches are not feasible.

## Case report

A 17-year-old female patient was admitted to the Obstetrics and Gynecology Department of the General Hospital of Trikala, Trikala, Greece, 7 days after undergoing a cesarean section. She presented with abdominal pain and fever accompanied by chills. This was her first cesarean delivery, performed at a private maternity clinic under prophylactic antibiotic coverage with cefotetan (Mefoxil^®^), administered as 2 g intraoperatively and an additional 2 g 12 h later. According to the attending obstetrician-gynecologist, the procedure was scheduled due to breech presentation and carried out via a typical Pfannenstiel technique, with no reported intraoperative or early post-operative complications. The patient reported progressive abdominal pain localized along the incision line over the preceding 3 days. A low-grade fever that began shortly after discharge (temperature peaked at 38.6˚C) was reported over the past 2 days, accompanied by chills. Blood tests revealed elevated white blood cells (21.9x10^3^/ml) which were predominantly neutrophils and increased levels of CRP (24.95 mg/dl), indicating an acute inflammatory response, with a post-cesarean section bacterial infection being the most probable cause. Testing also indicated the presence of a mild coagulation disorder, with elevated platelets (451x10^3^/ml) and markers of hemorrhagic diathesis (elevated international normalized ratio), abnormalities which were attributed to the effects of the acute inflammatory response (Table I). The results of urinalysis did not reveal any notable findings. The patient had no significant past medical history. She was classified as overweight, with a height of 172 cm, weight of 79 kg, and a BMI of 26.7.

**Figure 1:**
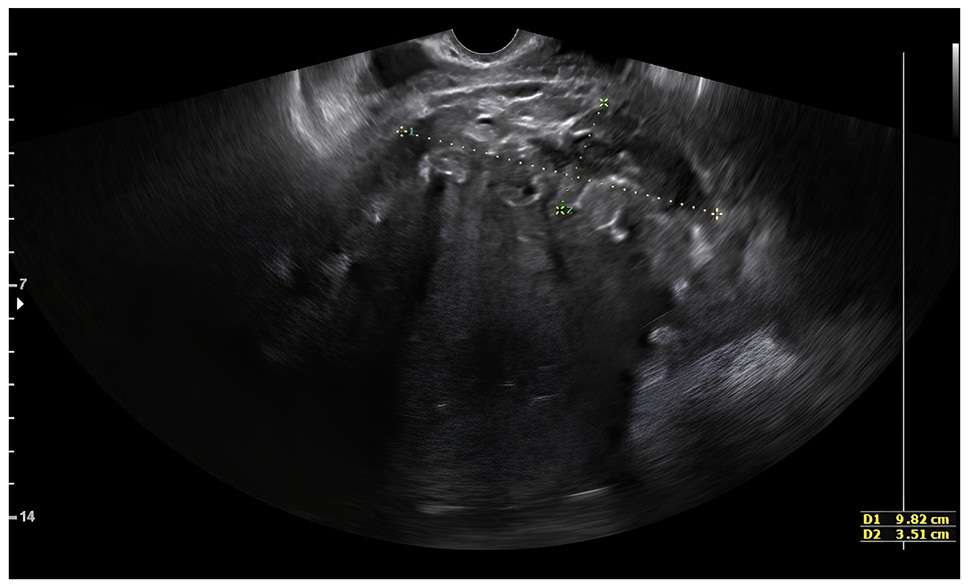
Transvaginal ultrasound image demonstrating a 9.8x3.5 cm mixed echogenic mass located in the pre-vesical space, at the level of the uterine scar, marked by two diameters.

**Figure 2:**
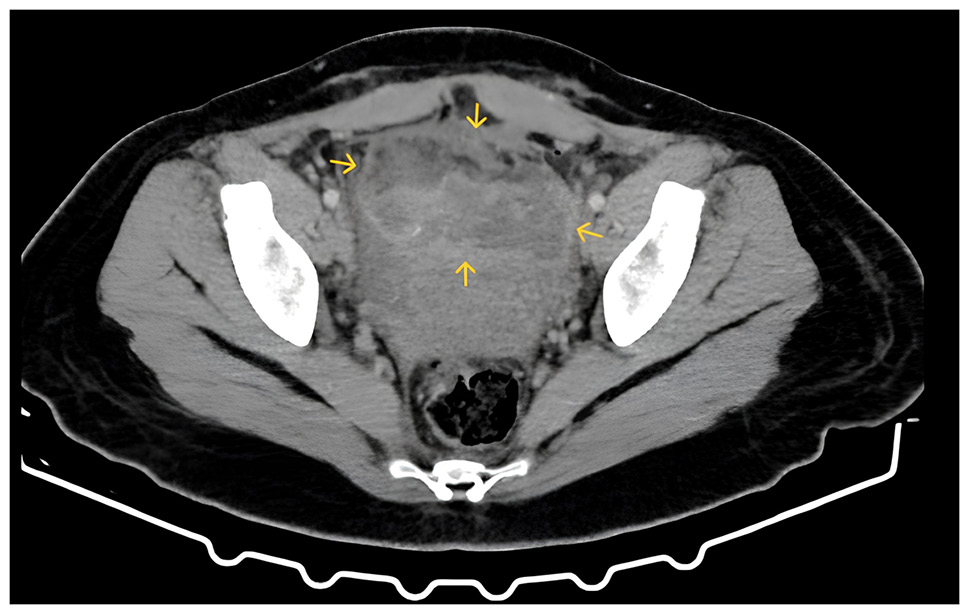
Computed tomography scan revealing a pelvic abscess at the uterine scar at the level of the isthmus (yellow arrows), characterized by a large, irregular, inhomogeneous solid-cystic mass with entrapped intralesional air bubbles.

A clinical examination revealed no signs of wound infection. The patient was febrile with a temperature of 38.3˚C, while her blood pressure was 110/70 mmHg and her heart rate was 77 bpm. Abdominal palpation elicited tenderness in the lower abdomen, although no peritoneal signs were present. A transvaginal ultrasound identified a mixed echogenic mass measuring 9.8x3.5 cm, located at the site of the uterine scar anterior to the bladder (Fig. 1). A subsequent computed tomography (CT) scan confirmed the presence of a large, irregular mass with solid-cystic components and intralesional air bubbles (Fig. 2). Empiric intravenous antibiotic therapy was initiated with piperacillin-tazobactam (Tazocin^®^) 4.5 g every 6 h and tigecycline (Tygacil^®^) at a 100-mg loading dose followed by 50 mg twice daily.

**Figure 3:**
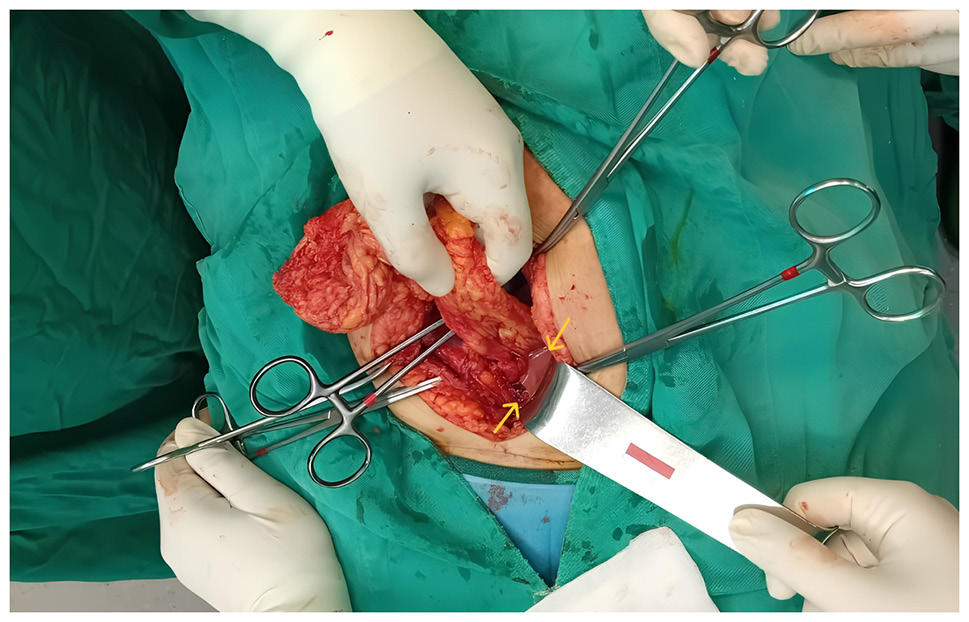
Intraoperative image illustrating inflamed omental adhesions and purulent collection at the site of the uterine scar (yellow arrows), consistent with the diagnosis of uterine scar abscess.

Following 48 h of intravenous antibiotic treatment without clinical improvement, and due to persistent symptoms and imaging findings, conservative management was considered insufficient and the decision for surgical intervention was made. A laparotomy was performed through the existing cesarean section incision. Intraoperatively, the omentum was found inflamed and adherent to the uterine wall at the scar site. Purulent material was identified adjacent to the uterine incision (Fig. 3). The abscess was drained, the area thoroughly irrigated, and a drainage tube was placed. Microbiological cultures of the purulent fluid isolated *Escherichia coli*. Antibiogram analysis revealed that the pathogen was resistant to ampicillin, amoxicillin-clavulanic acid, trimethoprim/sulfamethoxazole (co-trimoxazole), tetracycline, cefotaxime and ceftriaxone, while it presented sensitivity to piperacillin-tazobactam, imipenem, meropenem, tigecycline, amikacin, fosfomycin and nitrofurantoin. Since the isolated bacterium was sensitive to the antibiotics which were already being administered, piperacillin-tazobactam was continued for an additional 3 days post-operatively.

**Figure 4:**
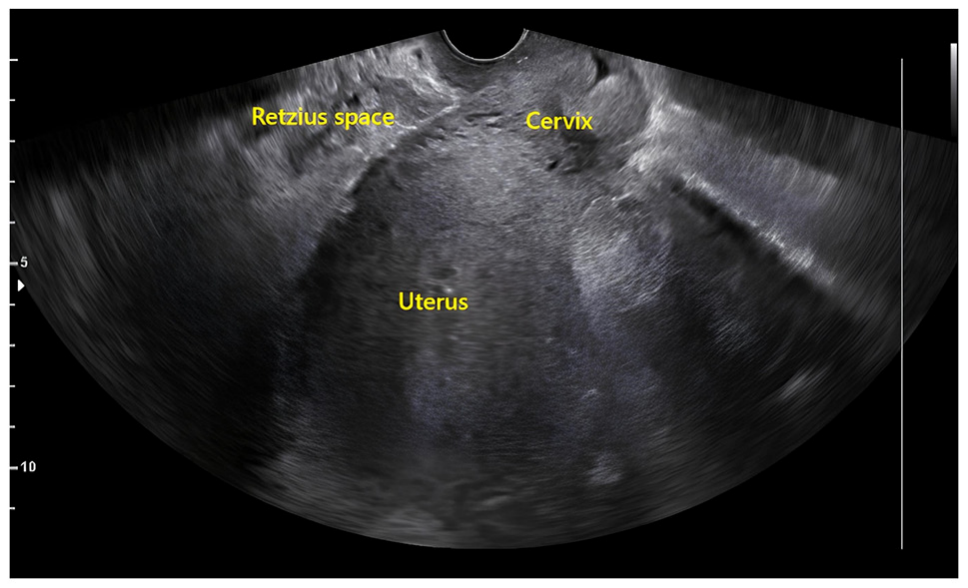
Follow-up transvaginal ultrasound performed 1 month following surgical drainage, illustrating the complete resolution of the abscess and restoration of normal pelvic anatomy.

The post-operative course was smooth. The patient was monitored daily via clinical examination, vital sign assessment and laboratory investigations throughout her stay at the hospital. She remained afebrile and exhibited normalization of inflammatory markers and coagulation profile as she recovered (Table I). No early post-operative complications were recorded during the patient's stay at our hospital. She was discharged in a good condition on the 5th day postoperatively. A follow-up transvaginal ultrasound performed 1 month thereafter and revealed no abnormalities (Fig. 4) and the patient reported no complications during that time. At the time of writing, at ~8 months since the discharge of the patient, she reports no persisting or recurrent symptoms pertaining to the abscess.

## Discussion

The present case report describes a rare case of cesarean scar abscess formation as an early complication of cesarean delivery. Clinical diagnosis of this condition may be challenging due to its non-specific presentation and symptomatology. In the case presented herein, clinical information alone only indicated abdominal infection, with symptoms, such as abdominal pain and fever, with or without chills. A similar presentation is described in the case report by Murayama *et al* (5), in which 2 patients also presented with simple abdominal pain and fever, without any more specific symptoms. More severe symptomatology may also be present, such as the case reported by Choden *et al* (8), where the patient had a high fever accompanied by tachycardia and signs of generalized peritonitis, along with pus discharge for the cesarean incision sites; these findings can better guide diagnosis, but can complicate management. Similar manifestations were also noted by the larger cohort study by Wu *et al* (9), who examined 23 cases of post-cesarean scar abscess in a 10-year period, cases that constituted a mere 0.182% of all women undergoing cesarean section at their institution, highlighting the rarity of the condition. All included women reported fever and abdominal pain, similar to the manifestations reported in the present case report (9).

Another factor that should be considered is the timing of the complication occurrence. In the present case report, along with the case reports by Murayama *et al* (5) (2 cases) and Choden *et al* (8), the abscess occurred within 7, 10, 6 and 4 days from the cesarean section, respectively, indicating early, acute manifestation. This was also corroborated by the findings presented in the study by Wu *et al* (9), where patients all exhibited symptoms within 30 days following delivery. However, several studies have also indicated another form of manifestation, in particular, isthmocele abscess several years after the performance of caesarean section (10-12). While this late-onset variant of the complication may not share the same underlying cause as the more acute form described in the present case report, it is perhaps indicative of an increased vulnerability to infection and purulence at this thinner and scarred region of the uterus.

Given the lack of specificity of clinical manifestations, the timely diagnosis of pelvic post-cesarean abscess is based upon imaging modalities, such as ultrasound, CT scan, or magnetic resonance imaging (MRI) (8). Transvaginal ultrasound constitutes the first-line imaging modality in Obstetrics and Gynecology, facilitating dynamic imaging of the pelvis at the outpatient level, which was the reason why it was the first modality employed in the case presented herein. This was also consistent with the diagnostic algorithm followed by Murayama *et al* (5) and Choden *et al* (8), and the methodology described in the cohort study by Wu *et al* (9), as all patients underwent transvaginal ultrasound to identify and measure the abscess. A CT scan constitutes another commonly used imaging modality for the investigation of the abdominal cavity and the diagnosis of serious postoperative complications after cesarean section, including pelvic abscess (13). A CT scan is generally preferred due to its widespread availability, lower costs and a shorter examination time in case of emergency; however, an MRI can serve as a valuable alternative when a CT scan is contraindicated or does not provide adequate information (14,15). In the present case report, transvaginal ultrasound initially revealed the presence of abnormality in the abdominal cavity and a CT scan was sufficient in diagnosing a mass with characteristics typical of abscess; therefore an MRI was unnecessary and was thus not performed. A CT scan was also sufficient for the diagnosis and treatment planning of the cases by Murayama *et al* (5) and Choden *et al* (8). An MRI was instead employed by Wu *et al* (9) in their cohort, along with ultrasonography in order to accurately measure the abscess. An MRI may also be appropriate in cases where additional concurrent abdominal pathologies exist and a more detailed investigation is warranted.

Currently, no standardized treatment protocol exists for pelvic abscess treatment following cesarean section. Initial, conservative management typically involves intravenous antibiotic therapy, which has a success rate of ~70% (16). In the absence of specific guidelines, in the case presented herein, a piperacillin-tazobactam and tigecycline regimen was used, with the aim of covering a wide spectrum of both Gram-positive and -negative bacteria. Initial empirical antibiotic regimen varied considerably in the available literature, with Murayama *et al* (5) utilizing ampicillin (4 g/day) plus gentamicin (160 mg/day) and clindamycin (1,200 mg/day) for 1 patient and (ampicillin (4 g/day) for the other one. Choden *et al* (8) utilized ceftriaxone (2 g intravenously) for their patient and continued the regimen following the confirmation of sensitivity, whereas Wu *et al* (9) administered most commonly, imipenem and cilastatin. What all regimens had in common, was their wide-spectrum action against both Gram-positive and -negative bacteria, since both types were likely to be the underlying cause; the former originating as contamination from the skin during surgery and the latter from urogenital and uterine epithelium. This was also proven by the large variety of pathogens reported; the most common pathogens isolated were Gram-negative (*Escherichia coli)*, as reported by Wu *et al* (9) and in the present case report, whereas Choden *et al* (8) reported a Gram-positive pathogen (*Staphylococcus aureus*).

In almost all cases however, conservative treatment failed (symptoms persisted) and surgery was thus performed. In appropriately selected cases, in which the abscess is not located in multiple locations (single), nor is it blocked by the intestine, uterus, or bladder, image-guided percutaneous drainage, via CT scan or ultrasound, is a less invasive alternative to surgery (17). Success rates for image-guided drainage with a CT scan and ultrasound are reported at 83.3 and 92% of cases, respectively (18,19), constituting and effective, minimally invasive alternative. However, as previously mentioned, performing image-guided drainage requires clear and direct access to the abscess, without the interposition of other organs and tissues, which is not always achievable. In fact, this was the reason why this treatment option was not employed by Murayama *et al* (5) in their cases and in the present case report, where percutaneous drainage was ruled out due to bowel loops in the pre-vesical space, making aspiration unsafe. In the presence of superficial abscess and wound dehiscence, simple debridement with or without abdominal wall incision and suturing may be employed, which was the case for the majority of patients reported by Wu *et al* (9). This option was not feasible for the case presented herein, due to the depth of the abscess and the absence of any signs of superficial purulence at the incision site. Laparoscopy constitutes a common modality for minimally invasive pelvic abscess treatment, with lower complication rates, reduced postoperative pain, and shorter hospital stay (20,21). This method was used by Murayama *et al* (5), with both patients recovering well and one successfully delivering again 22 months following the initial surgery. However, laparoscopy may be limited by factors such as intra-abdominal adhesions or bowel distension, rendering its application challenging or unsafe. Furthermore, in cases of exacerbating patient clinical condition, laparotomy may be the treatment of choice, facilitating faster and more thorough intervention, as was the case in the report by Choden *et al* (8). In the present case report, laparoscopy was not performed due to unavailability; therefore, laparotomy through the cesarean incision was considered the most appropriate feasible option.

Even more important than treatment is the prevention of such conditions in the first place. Infection and abscess formation following cesarean delivery may be caused by a variety of factors, related to the mother (younger or older age, obesity, rural residence, pre-gestational diabetes, a history of prior cesarean section); the pregnancy (hypertensive disorder, gestational diabetes, twin pregnancy, premature rupture of membranes, prolonged trial of labor prior to cesarean section, chorioamnionitis); and the delivery itself (emergency cesarean section, the absence of antibiotic prophylaxis, longer duration of surgery and transfusion) (22). In the present case report, the extreme young age of the patient (17 years) may have been the factor that most likely led to the occurrence of this complication. With knowledge of the risk factors, certain prevention strategies may be formed in order to minimize the chances that similar complications occur in the future. In particular, improved perioperative practices, via adequate diabetes control, skin preparation (shaving, disinfection), vaginal preparation and antibiotic prophylaxis; intra-operative practices such as operating room staff training and oxygen supplementation and post-operative assessment with daily inspection and laboratory investigations are effective ways to reduce the incidence of this complication and subsequently patient post-partum morbidity and mortality (22).

In conclusion, pelvic abscess at the uterine scar following cesarean section is a rare, yet severe complication, associated with increased maternal morbidity and mortality, despite the routine use of prophylactic antibiotic. When conservative treatment fails, and image-guided drainage is not feasible or laparoscopy is unavailable, laparotomy remains the definitive and effective surgical intervention. However, the conclusions extracted from the management of this single case may not be applicable to all clinical settings and larger, multi-center cohort studies or reporting registries are necessary in order to establish optimal diagnosis and management algorithms for this rare complication.

## Figures and Tables

**Table I:** Laboratory tests of the patient during her hospitalization at the Obstetrics and Gynecology Clinic of the General Hospital of Trikala (Trikala, Greece).

Laboratory tests	Day of admission to the clinic	2nd Day of hospitalization	1st Post-operative day	2nd Post-operative day	5th Post-operative day	Normal laboratory values
Ht	30.1%	29.2%	28.1%	28.6%	28.9%	37.7-49.7%
Hb	9.6 gr/dl	9.4 gr/dl	9.1 gr/dl	9.2 gr/dl	9.2 gr/dl	11.8-17.8 gr/dl
PLT	451x10^3^/ml	476x10^3^/ml	318x10^3^/ml	256x10^3^/ml	225x10^3^/ml	150-350x10^3^/ml
WBC	21.9x10^3^/ml	20.8x10^3^/ml	19.1x10^3^/ml	13.7x10^3^/ml	7.6x10^3^/ml	4-10.8x10^3^/ml
NEUT	94.7%	93.3%	89.2%	87.2%	66.3%	40-75%
CRP	24.95 mg/dl	25.17 mg/dl	18.23 mg/dl	9.15 mg/dl	1.06 mg/dl	<0.7 mg/dl
APTT	35.7 sec	35.8 sec	33.1 sec	31.3 sec	29.8 sec	24.0-35.0 sec
INR	1.31	1.44	1.35	1.27	1.11	0.8-1.2
FIB	207 mg/dl	201 mg/dl	211 mg/dl	225 mg/dl	234 mg/dl	200-400 mg/dl
Glu	125 mg/dl	91 mg/dl	83 mg/dl	81 mg/dl	-	75-115 mg/dl
Cr	0.85 mg/dl	0.71 mg/dl	0.65 mg/dl	0.67 mg/dl	-	0.40-1.10 mg/dl
κ^+^	3.38 mmol/l	3.94 mmol/l	4.01 mg/dl	3.98 mg/dl	-	3.5-5.1 mmol/l
Να^+^	132.6 mmol/l	137.8 mmol/l	138.9 mmol/l	141.8 mmol/l	-	136-145 mmol/l
TBIL	0.58 mg/dl	-	-	-	-	0.3-1.2 mg/dl
SGOT	27 IU/l	-	-	-	-	5-33 IU/l
SGPT	18 IU/l	-	-	-	-	10-37 IU/l
AMY	38 IU/l	-	-	-	-	30-118 IU/l

Ht, hematocrit; Hb, hemoglobin; PLT, platelets; WBC, white blood cells; NEUT, neutrophils; CRP, C-reactive protein; APTT, activated partial thromboplastin time; INR, international normalized ratio; FIB, fibrinogen; Glu, glucose; Cr, creatinine; K^+^, potassium; Na^+^, sodium; TBIL, total bilirubin; SGOT, serum glutamic oxaloacetic transaminase; SGPT, serum glutamate pyruvate transaminase; AMY, amylase.

## Data Availability

The data generated in the present study may be requested from the corresponding author.
